# Prescription writing quality of psychotropic agents in MBBS interns in a private medical college hospital in Mangaluru

**DOI:** 10.12688/f1000research.144712.1

**Published:** 2024-04-15

**Authors:** Poorva Bakshi, Sharanya B. Shetty, Shreya Bera, Priyanka Renita D'Souza, Keshava Pai

**Affiliations:** 1 Kasturba Medical College, Mangalore, Manipal Academy of Higher Education, Manipal, India; 2 Department of Psychiatry, Kasturba Medical College, Mangalore, Manipal Academy of Higher Education, Manipal, India

**Keywords:** Prescription, intern, rational prescription writing, Quality Improvement Project, Audit, Psychiatry

## Abstract

**Background:**

Rational prescription writing is an important skill to master during internship. This Quality Improvement (QI) project aimed to understand the state of prescription writing among interns posted in the Department of Psychiatry, analyze the causes responsible for errors in prescription writing and bring about a change in the current practice.

**Methods:**

The MBBS interns are posted in the Department of Psychiatry for 15 days. During day 1 to day 5 of their posting, a pre intervention phase was conducted wherein prescriptions written by interns in the Department of Psychiatry were collected. The prescriptions were scored based on 14 criteria which were selected based on World Health Organization (WHO) guidelines and Medical Council of India (MCI) ideal prescription format. During PDSA (Plan Do Study Act) Cycle 1, an educational handout was distributed to the interns containing the MCI ideal prescription format and WHO guidelines regarding prescription writing. The brochure was also verbally explained to the interns. From day 7 to day 15 of their posting, prescriptions written by the interns were collected. The prescriptions were scored using the same criteria.

**Results:**

During the pre intervention phase the mean total score of prescriptions was 9.54 ± 1.003. There was a significant improvement in the mean total score to 10.26 ± 0.746. There was a 7.54% improvement. There was also a significant improvement in several individual criteria.

**Conclusions:**

The first PDSA cycle was successful in improving the quality of prescription writing among interns posted in the Department of Psychiatry. There is a need to implement more PDSA cycles to improve the quality still further.

## Introduction

The problem of irrational drug prescription is prevalent worldwide. ^1^ According to a World Health Organization (WHO) estimate, more than 50% of drugs are prescribed, dispensed, or sold inappropriately, and 50% of patients fail to take them correctly ( https://www.who.int/activities/promoting-rational-use-of-medicines).

The prescription is the written instruction of the physician communicated to the patient. Writing rational prescriptions is an important skill for physicians. Poorly written prescriptions can negatively affect patient treatment, including worsening of the disease and making the prescriber liable to influence, which may lead to irrational prescribing. ^2^

Rational prescribing requires a logical approach that involves several key steps, including making the diagnosis, determining the therapeutic goals, consideration of treatment options, deciding the best treatment option, prescription and monitoring. ^3^

In clinical practice, a doctor must be able to diagnose and immediately decide on the drug to be prescribed. Furthermore, patients may have different age, sex, and sociocultural features that affect the drugs prescribed.

Internship is a period of one year during which newly qualified Bachelor of Medicine and Bachelor of Surgery (MBBS) graduates practice under the guidance of senior doctors in designated hospitals. Studying the pattern of their prescriptions and suggesting changes is of utmost importance in improving the quality of patient care and enhancing the undergraduate medical education of interns.

### Available knowledge

Most studies on the impact of medical errors, including prescription writing errors, have been conducted in developed countries. Medical errors are a leading cause of mortality in the United States. Studies conducted in Asian countries have reported varying rates of errors when writing prescriptions. These values range from 7% to 35.4%. ^4^

A study conducted in Bangladesh found that 78% of drugs were prescribed by their generic names, 85% complied with the essential drug list, and 81% were dispensed according to prescription. ^5^

In a study conducted in private hospitals in western India, prescriptions were analyzed according to WHO prescribing indicators. It was found that out of the 250 prescriptions analyzed, patient details were not written in 100% of prescriptions. Drug dosage, instructions regarding drug administration, and the duration of the treatment were not completely written in 90%, 74%, and 80% of prescriptions studied, respectively. The prescriber’s medical registration number was not mentioned in any of the prescriptions. ^6^

In a study carried out in the non-governmental organization sector in West Bengal, India, it was found that the majority were signed, legible, and complete with respect to age/gender data, 95.5% used Latin abbreviations, and 7.7% mentioned neither signs, symptoms, nor diagnosis. Irrational fixed-dose combinations were used in 45.6% of the prescribed drugs. Only 45.7% of prescribed drugs conformed to the WHO model list of essential drugs. ^7^

In a study of selected rural pharmacies in southern India, it was found that 91.4% of prescriptions did not contain any drug prescribed by its generic name. ^8^

In a study in The Oxford Dental College and Hospital, Bangalore, prescriptions were analyzed for several features, such as patient details, doctor’s information, and drug information. Prescriptions written by undergraduate students were better than those written by interns and postgraduates (PGs). ^9^

The prescriptions written by interns were studied in a primary health center in India, and it was found that 34.97% of drugs were prescribed by generic name and 58.7% of drugs prescribed were from the essential drug list of India. ^10^

### Rationale

After studying the literature available regarding the state of prescription writing, this Quality Improvement (QI) project was conceptualized. This QI project sought to understand and bring about significant improvements in the quality of prescriptions written by interns posted in the Department of Psychiatry.

**Figure 1.**
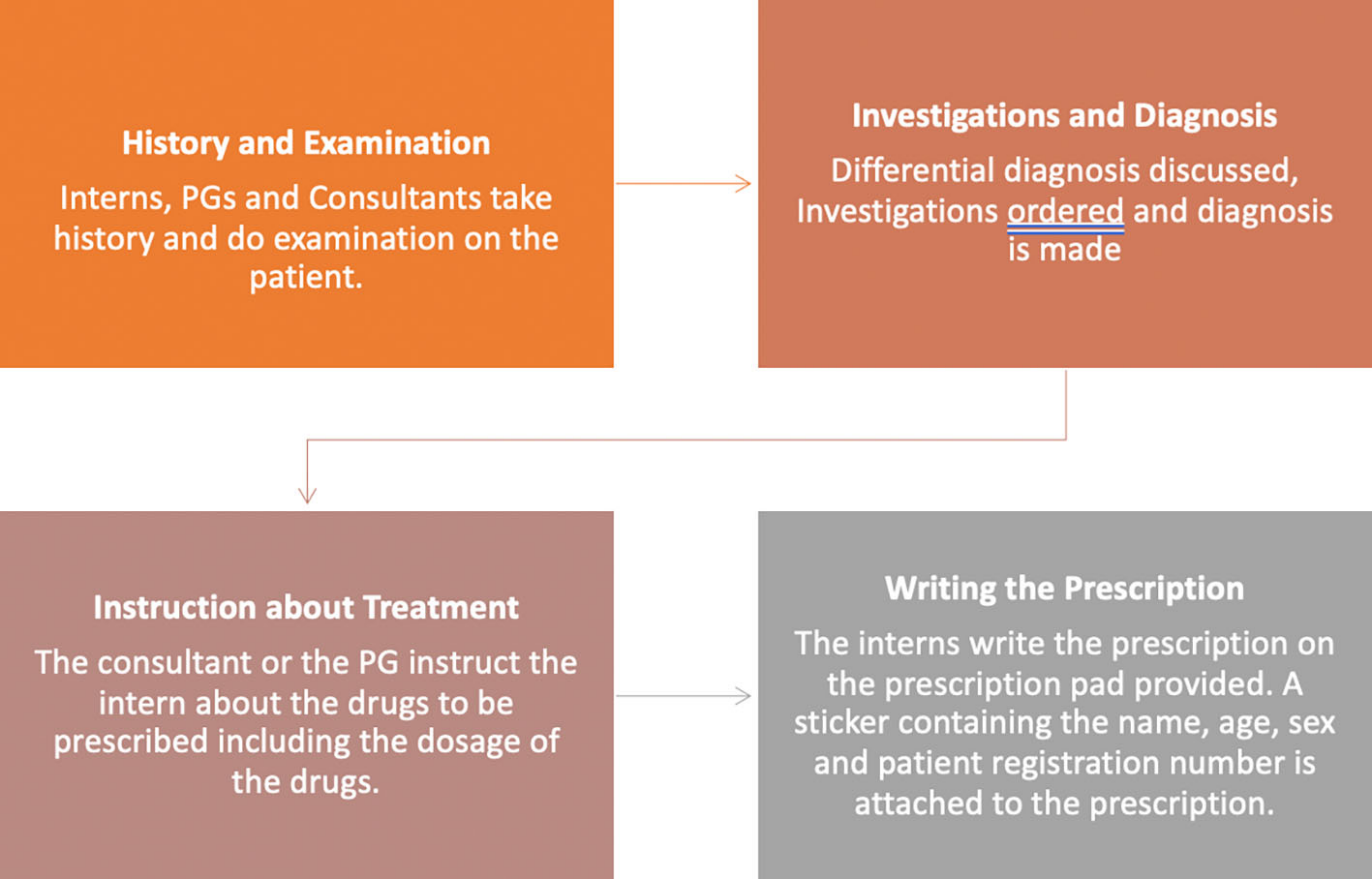
Process map regarding the process by which interns write prescriptions.

A process map was created that outlined the process through which the interns were posted in the Department of Psychiatry to write prescriptions ( Figure 1).

**Figure 2.**
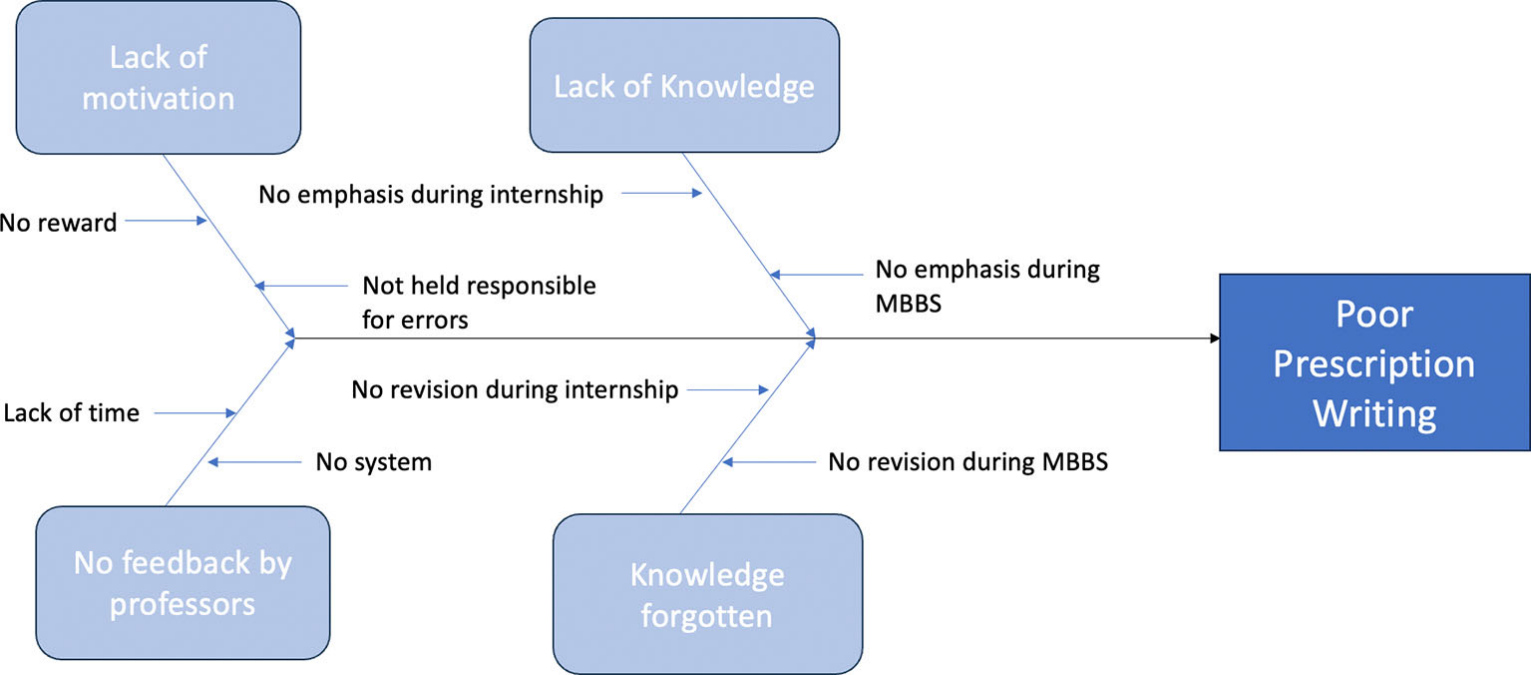
Fishbone diagram regarding causes of poor prescription writing.

We carefully studied the process and brainstormed possible causes of the poor quality of prescription writing among interns with the help of a fishbone diagram ( Figure 2).

### Aims

The aim of our QI project was to improve the quality of prescription writing among interns in the psychiatry department.

Our Specific, Measurable, Applicable, Realistic and Timely (SMART) aim was to improve the mean prescription scores.

## Methods

**Study setting:** This study was conducted in the outpatient Department of Psychiatry, Kasturba Medical College Hospital, Attavar, Mangaluru.

**Study design:** Quality Improvement Project.

**Study participants:** MBBS interns in the Department of Psychiatry, Kasturba Medical College Hospital, Attavar, Mangaluru.

### Inclusion criteria

a) MBBS Interns posted in the Department of Psychiatry, willing to grant consent.
b) Patients above the age of 18 years attending Psychiatry Outpatient department.

### Exclusion criteria

a) Postgraduates and consultants writing prescriptions for the patients
b) Interns posted in other specialties

**Study duration:** 1 year from November 2022 till August 2023

### Sample size

The sample size for the study is 85 with 5% level of significance.

$$\begin{array}{l} N=Z^{2}\mathrm{\rho v}/d^{2}\\ \;={\left(1.96\right)}^{2}\left(0.67\right)\left(1-0.67\right)/{\left(0.1\right)}^{2}\\ \;=85 \end{array}$$

Z = 1.96 is standard normal value at 5% level of significance

Ρ = Proportion = 67%

d = Absolute Precision = 10%

**Sampling method:** Convenient sampling method.

**Methodology:** As a part of compulsory rotation during internship, the interns are posted in the Psychiatry department for 15 days. We used the Plan-Do-Study-Act (PDSA) model to conduct this Quality Improvement Project. The PDSA model is a four-step model used to improve the process or bring about a change in a system. We divided our study into two phases. The pre intervention phase and PDSA cycle 1 were repeated for each batch of interns who participated in this study.

The PDSA model used for this study consisted of the following:

Plan: Based on the results of the pre intervention phase, we planned to implement the intervention.

Do: We implemented the intervention

Study: We studied the result of the intervention

Act: Using an educational handout on prescription writing during internship

### Pre intervention phase

On the first day of the psychiatry postings, the interns were informed about the study and written informed consent was obtained from them. They were instructed to create copies of the prescriptions that they wrote with the help of a carbon sheet which was provided with the empty prescriptions. Informed written consent was obtained from the patients whose prescriptions were used in the audit.

For the first 5 days of Psychiatry posting, carbon copy of the prescriptions were collected before giving the original prescription to the patients.

### PDSA cycle 1

On day 6 of the posting, the interns were given an educational handout which was based on the WHO practical manual guide to good prescribing that explained the format of the prescriptions to be followed. The investigators also explained regarding the format of the prescription writing to be followed by the interns.

On days 7–15, the interns again created copies of the prescriptions they were writing with the help of carbon sheets provided with the empty prescriptions, and these were collected before giving the original prescription to the patients.

### Tool for data collection

a) **Criteria for assessing the quality of prescription:** To determine the quality of prescription writing, we established certain criteria. These were based on WHO guidelines. ^11^ The Medical Council of India (MCI) ideal prescription format was also used to create the criteria ( https://delhimedicalcouncil.org/images/Latest-News/modalprescription.pdf). The prescriptions were evaluated using the following criteria: If the criteria are present, 1 point will be awarded, and if the criteria are absent, 0 points will be awarded. Then, the final score of that prescription was calculated by adding the points. The scores range from 0 to 14.
  1. Date
  2. Name of patient
  3. Age of patient
  4. Contact number of the patient
  5. Gender of the patient
  6. Generic name of the drug
  7. Drug name in capital letter
  8. Strength
  9. Dosage form
  10. Route of Administration
  11. Total quantity of drugs to be given by pharmacy
  12. Number of days to take the drug
  13. Number of times to take the drug in a day
  14. Signature or initials of the prescriber
b) **Educational handout:** This was based on the WHO Practical Manual Guide to Good Prescribing. ^11^

**Biological materials required - none**

### Data analysis

Statistical analysis was performed using IBM SPSS Statistics for Windows version 29 (IBM Corp., Armonk, N.Y., USA). ^12^ The copyright license has been obtained by the institution. An open source alternative is Jamovi version 1.x. ^13^ Means and standard deviations were calculated. The t-test was used for continuous variables, and the chi-square test was used for categorical variables. Statistical significance was set at p value<0.05.

### Pre-registered data analysis

The data analysis in this study was not pre-registered.

## Results

### Pre intervention phase results

In total, 91 prescriptions were analyzed. The mean score of prescriptions written by the interns was 9.54 ± 1.003. The mean scores across the various criteria were as follows ( Table 1): ^17^

**Table 1.** Mean scores of participants in pre intervention phase.

	Mean	Std. Deviation
Date	0.68	0.469
Name of Patient	1.00	0.000
Age of Patient	0.97	0.180
Contact Number of the Patient	0.00	0.000
Gender of the Patient	0.93	0.250
Generic Name of the Drug	0.19	0.392
Drug Name in Capital Letter	0.96	0.206
Strength	0.86	0.352
Dosage form	1.00	0.000
Route of Administration	0.00	0.000
Total quantity of drugs to be given by pharmacy	0.08	0.268
Number of days to take the drug	0.95	0.229
Number of times to take the drug in a day	0.98	0.147
Signature or initials of the prescriber	0.96	0.206

Std.- Standard.

### PDSA cycle 1 results

A total of 84 prescriptions were analyzed. The mean score of prescriptions written by the interns was 10.26±0.746. The mean scores across the various criteria were as follows ( Table 2):

**Table 2.** Mean scores of participants in PDSA Cycle 1.

	Mean	Std. Deviation
Date	0.96	0.187
Name of Patient	1.00	0.000
Age of Patient	1.00	0.000
Contact Number of the Patient	0.00	0.000
Gender of the Patient	1.00	0.000
Generic Name of the Drug	0.04	0.187
Drug Name in Capital Letter	1.00	0.000
Strength	0.90	0.295
Dosage form	0.92	0.278
Route of Administration	0.02	0.153
Total quantity of drugs to be given by pharmacy	0.49	0.503
Number of days to take the drug	0.94	0.238
Number of times to take the drug in a day	1.00	0.000
Signature or initials of the prescriber	0.99	0.109

Std.- Standard.

There was a significant improvement in the mean total score from 9.54±1.003 to 10.26±0.746 (p<0.01). There was an improvement of 7.54% after PDSA cycle 1.

There was also a significant improvement in the following criteria: date (p<0.01), sex of patient (p=0.13), generic name of the drug (p=0.01), drug name in capital letters (p=0.045), dosage form (p=0.07), and total quantity of drugs to be administered by the pharmacy (p<0.01).

There was no significant improvement in the Age of Patient (p=0.083), strength (p=0.336), route of administration (p=0.159), number of days to take the drugs (p=0.897), number of times to take the drug in a day (p=0.174), or signature or initials of the prescriber (p=0.196).

## Discussion

In this QI project, we attempted to ascertain methods to improve the quality of prescriptions written by interns posted in the Department of Psychiatry.

In the first PDSA cycle, we tried to fill in the knowledge gap with the help of a brochure and a verbal explanation of the format of prescription writing as prescribed by the MCI as well as the guidelines stipulated by the WHO.

After PDSA cycle 1, we found a significant improvement in the total scores of the prescriptions as well as a significant improvement across several criteria, including date, gender of patient, generic name of the drug, drug name in capital letters, dosage form, and total quantity of drugs to be given by the pharmacy.

The aim of this QI project was to improve prescription writing scores. After the first cycle, a 7.54% improvement was observed.

A study conducted in The Oxford Dental College and Hospital, Bangalore, found that the patient’s name (95.6%), age (91.2%), and gender (91.6%) were written by the majority of the students. The patients’ outpatient number (0%), address (0%), and contact number (0.4%) were missing in almost all prescriptions, which is in agreement with our study where the name, age, and gender were mentioned in 100% of the prescriptions collected. ^9^

Furthermore, a study was conducted regarding the impact of patient-based teaching in improving the prescription writing skills of II MBBS students. This study showed that patient-based teaching improves responsibility, focus, and memory. ^14^

In our study, our informal teaching session at the end of PDSA cycle 1 revealed a similar improvement, which may be attributed to the orientation given.

In another study carried out at Nobel Medical Teaching Hospital, Biratnagar, Nepal, there was a 0% error in the patient’s name, age, and sex, which was similar to our findings. ^15^

However, the findings of other studies are different from ours. In a study conducted at Oxford Dental College and Hospital, Bangalore, it was found that the parameters that were lacking the most were dosage instructions, strength of the drugs, and duration of the drugs, which was not the case in our study. ^9^ According to our study, the total quantity of drugs to be given by the pharmacist, dosage form, and generic names were not mentioned.

The lack of generic names written in our study could be because interns have very little time to write prescriptions or because psychiatric medications are not as common as analgesics or gastritis medicines; hence, additional work and reading is required to become aware of them, and either the interns did not have an incentive to learn this or did not have enough time to cover this aspect of prescription writing.

In our study, the criteria for which there was no significant improvement were criteria that already had high scores in the pre intervention phase.

Furthermore, another study in Patna, where prescriptions from different OPDs in different departments were audited, showed that only 10% of the prescriptions contained drugs prescribed in their generic name. Frequency, route, and duration of drug administration were mentioned in 86.7%, 79.3%, and 69.6% of the prescriptions, respectively. Among the prescriptions, 42.5% were easily legible. Hence, it was not in accordance with the WHO standards. ^16^

Legibility was not a criterion for this study. If we had to look at it subjectively, none of the prescriptions were difficult to read by the people undertaking the study, thus giving us a higher score in that field than in the study mentioned above.

An important criterion that was judged in the study conducted in Nobel Medical Teaching Hospital, Biratnagar, were 2 same drugs or the same category mentioned in the same prescription, which accounted for 0.5% of their total errors. ^15^ This category was not judged in our study because it was not a criterion mentioned in the MCI ideal prescription and it is an indicator of skills and not methodology, which was what our study mostly focused on.

### Limitations

Instructing interns to make copies of their prescriptions may have led them to pay closer attention to their prescription writing, leading to a biased result.

## Conclusion

This Quality Improvement project enabled us to understand that brochures regarding prescription writing and verbal explanations are useful tools for improving the quality of prescriptions written by interns in the department of psychiatry. Therefore, there is a need to find more innovative methods to improve the quality of prescriptions. There is a need to improve interns’ training by emphasizing rational prescription writing during internships. Further studies are required to determine whether this method is universally applicable.

### Ethics and consent

The protocol was approved by the Institutional Ethics Committee Kasturba Medical College, Mangalore (Reg. No. ECR/541/Inst/KA/2014/RR-20) before commencement of the Quality Improvement Project and Ethical Approval was obtained. The protocol number of the study was IECKMCMLR-10/2022/423. The date of approval was 19/10/2022. Written informed consent for the study was obtained from the interns and the patients/relatives of the patients, respectively. Participants had the right to withdraw from the study at any time.

## Data Availability

Open Science Framework: Prescription writing quality of psychotropic agents in MBBS interns at a private medical college hospital in Mangaluru. https://doi.org/10.17605/OSF.IO/HZGFC. ^17^ This project contains the following data:
• Intern prescription writing scores xlsx (scores of prescriptions written by interns during the preintervention phase and PDSA Cycle 1). • Educational Handout.docx (the educational handout given to interns during PDSA Cycle 1). • Intern Consent Form.docx • Patient Consent Form.docx • Checklist for Prescription writing quality of psychotropic agents in MBBS interns in a private medical college hospital in Mangaluru.docx This study was based on the SQUIRE (Standards for Quality Improvement Reporting Excellence) guidelines. ^18^ Data are available under the terms of the Creative Commons Attribution 4.0 International license (CC-BY 4.0).
