# Supplementary material for: Prescriptions written by MBBS Interns in the Department of Psychiatry in a Medical College Hospital in Mangaluru: A Quality Improvement Study
Source: F1000Res. 2024 Dec 20;13:269. Originally published 2024 Apr 15. [Version 2] doi: 10.12688/f1000research.144712.2 (PMC11375400; doi:10.12688/f1000research.144712.2)
Supplement: Peer review history

# Reviewer report: Reviewer response for version 2

**DOI:** 10.5256/f1000research.176005.r351347

**Published:** 2025-01-30

**Authors:** Dr Shashwath Sathyanath

**Affiliations:** 1 Yenepoya Medical College, Mangalore, India

The article has been revised as per the earlier comments and appropriate changes have been made. This article may be approved for indexing.

Is the work clearly and accurately presented and does it cite the current literature?

Yes

If applicable, is the statistical analysis and its interpretation appropriate?

Partly

Are all the source data underlying the results available to ensure full reproducibility?

Yes

Is the study design appropriate and is the work technically sound?

Yes

Are the conclusions drawn adequately supported by the results?

Yes

Are sufficient details of methods and analysis provided to allow replication by others?

Partly

Reviewer Expertise:

Deaddiction psychiatry, Consultation-liaison Psychiatry, Mood Disorders.

I confirm that I have read this submission and believe that I have an appropriate level of expertise to confirm that it is of an acceptable scientific standard.

# Reviewer report: Reviewer response for version 2

**DOI:** 10.5256/f1000research.176005.r351348

**Published:** 2024-12-26

**Authors:** Vandana Gaur

**Affiliations:** 1 Department of Psychology, SDM Government PG College in Doiwala, Dehradun, Uttarakhand, India

The revised version is approved for indexing.

Is the work clearly and accurately presented and does it cite the current literature?

Yes

If applicable, is the statistical analysis and its interpretation appropriate?

Yes

Are all the source data underlying the results available to ensure full reproducibility?

Yes

Is the study design appropriate and is the work technically sound?

Yes

Are the conclusions drawn adequately supported by the results?

Yes

Are sufficient details of methods and analysis provided to allow replication by others?

Yes

Reviewer Expertise:

Human Psychology

I confirm that I have read this submission and believe that I have an appropriate level of expertise to confirm that it is of an acceptable scientific standard.

# Reviewer report: Reviewer response for version 1

**DOI:** 10.5256/f1000research.158550.r267459

**Published:** 2024-09-12

**Authors:** Sanjeev Badiger

**Affiliations:** 1 A J Institute of Medical Sciences and Research Centre, Mangaluru, India

The topic selected by the author is relevant to the present scenario , as prescription  is considered as an important  Document. This article highlights the need for quality prescription writing and the need for improvement in prescription writing . Studying the pattern of their prescriptions and suggesting changes is of utmost importance in improving the quality of patient care and enhancing the undergraduate medical education of interns.

 Major Points :

 Methodology : study sample size and study design is appropriate. But the capture other factors influencing the prescription pattern can be captured by adding qualitative component like FGD is helpful to improve the quality of the study  

 Minor points :

 Topic : Relevant and well phrased 

 Introduction: Adequate and highlighted the need for the study 

 Data analysis ,Discussion and conclusions are adequate 

 This research article can be accepted for indexing.

Is the work clearly and accurately presented and does it cite the current literature?

Yes

If applicable, is the statistical analysis and its interpretation appropriate?

Yes

Are all the source data underlying the results available to ensure full reproducibility?

Yes

Is the study design appropriate and is the work technically sound?

Partly

Are the conclusions drawn adequately supported by the results?

Yes

Are sufficient details of methods and analysis provided to allow replication by others?

Yes

Reviewer Expertise:

Communicable diseases and behavioral science

I confirm that I have read this submission and believe that I have an appropriate level of expertise to confirm that it is of an acceptable scientific standard.

# Response

**Published:** 2024-12-14

**Authors:** Sharanya Shetty

**Affiliations:** Psychiatry, Kasturba Medical College, Mangalore, Manipal Academy of Higher Education, Mangalore, Karnataka, India

Dear Sir,

 Thank you for your valuable feedback on our manuscript. We appreciate your insightful comments and suggestions, which has undoubtedly contributed to improving the quality of our manuscript. We have tried our best to address your concerns in the modified manuscript.

 We understand that focused group discussion would have added further insights on other qualitative aspects, which we will plan to incorporate in the next cycle. We have added the same as a limitation of our study.

 Thank you again for your valuable feedback.

 Regards

 Dr Sharanya B Shetty

# Reviewer report: Reviewer response for version 1

**DOI:** 10.5256/f1000research.158550.r313883

**Published:** 2024-09-04

**Authors:** Dr Shashwath Sathyanath

**Affiliations:** 1 Yenepoya Medical College, Mangalore, India

Summary of the article

 This Quality Improvement (QI) project aimed to enhance the quality of prescription writing among MBBS interns in the Department of Psychiatry at Kasturba Medical College Hospital, Mangaluru. The study addressed the prevalent issue of irrational drug prescriptions, which contributes to adverse patient outcomes and inefficiencies in healthcare. The project involved a pre-intervention assessment of prescription quality, followed by an intervention based on educational materials and feedback, and then a post-intervention assessment to measure improvements.

 Detailed Review

 1. Work Presentation and Literature Citation

    Response: Yes

    Summary: The work is presented clearly and logically. The introduction provides a comprehensive background on the problem of irrational drug prescribing and cites relevant literature. However, there is scope of citing few newer literatures from the studies in the last 3 years

 2. Study Design and Technical Soundness

    Response: Yes

    Summary: The study design is appropriate for the Quality Improvement (QI) project, utilizing the Plan-Do-Study-Act (PDSA) model. The project addresses a clear problem, implements an intervention, and evaluates its effectiveness in a systematic manner. However, the title of the article could mention the type of study design for the ease of the readers.

 3. Replication Details

    Response: Yes

    Summary : The methods and analysis are described in sufficient detail to allow replication.

   The article includes detailed descriptions of the intervention (educational handouts and verbal explanations), the assessment criteria for prescription quality, and the statistical methods used for analysis. The process maps and fishbone diagrams are provided, facilitating replication of the study.

 4. Statistical Analysis

    Response: Partly

    Summary: The statistical analysis is mostly appropriate but could benefit from some improvements.

   The use of means, standard deviations, t-tests, and chi-square tests is generally suitable. However, it may be beneficial to include additional analyses or sensitivity checks to confirm the robustness of the findings. In addition, the tables mentioned are not complete as it lacks the p values mentioned in the writings.

 5. Availability of Source Data

    Response: Yes

    Summary: The source data underlying the results are available for full reproducibility.

   The data, including prescription writing scores and the educational handout, are accessible via the Open Science Framework link provided. This transparency supports reproducibility and verification of the study’s findings.

 6. Support for Conclusions

    Response: Yes

    Summary: The conclusions are adequately supported by the results.

    The study demonstrates a significant improvement in prescription quality post-intervention, with specific criteria showing notable enhancements. The discussion contextualizes these findings within existing literature, reinforcing the study’s conclusions.

 Recommendations

 This study can be considered for indexed after a few minor corrections.

Is the work clearly and accurately presented and does it cite the current literature?

Yes

If applicable, is the statistical analysis and its interpretation appropriate?

Partly

Are all the source data underlying the results available to ensure full reproducibility?

Yes

Is the study design appropriate and is the work technically sound?

Yes

Are the conclusions drawn adequately supported by the results?

Yes

Are sufficient details of methods and analysis provided to allow replication by others?

Partly

Reviewer Expertise:

Deaddiction psychiatry, Consultation-liaison Psychiatry, Mood Disorders.

I confirm that I have read this submission and believe that I have an appropriate level of expertise to confirm that it is of an acceptable scientific standard.

# Response

**Published:** 2024-12-14

**Authors:** Sharanya Shetty

**Affiliations:** Psychiatry, Kasturba Medical College, Mangalore, Manipal Academy of Higher Education, Mangalore, Karnataka, India

Dear Sir,

 Thank you for your valuable feedback on our manuscript. We appreciate your insightful comments and suggestions, which has undoubtedly contributed to improving the quality of our manuscript. We have tried our best to address your concerns in the modified manuscript.

1. Taking your feedback into consideration, we have included newer studies in the introduction section.
2. We have also changed the title of the study to accurately reflect the study design.
3. We have merged our tables and also included p value in the table as per your suggestion.

Thank you again for your valuable feedback.

  Regards

 Dr Sharanya B Shetty

# Reviewer report: Reviewer response for version 1

**DOI:** 10.5256/f1000research.158550.r313887

**Published:** 2024-08-16

**Authors:** Vandana Gaur

**Affiliations:** 1 Department of Psychology, SDM Government PG College in Doiwala, Dehradun, Uttarakhand, India

The introduction provides a clear rationale for the study, emphasizing the importance of accurate prescription writing in clinical practice, particularly for psychotropic agents, which are associated with complex dosing and potential side effects. The study's objectives are well-defined, focusing on evaluating the prescription writing quality of psychotropic agents among MBBS interns. The results section (assuming it is well-presented) likely provides valuable insights into the quality of prescription writing among interns. It is essential that this section includes data on common errors and areas where interns perform well, as this information could inform future training programs. The discussion should ideally interpret the results in the context of existing literature, highlighting both strengths and weaknesses in the prescription writing practices of the interns. It should discuss the implications for patient safety and medical education.

 The study addresses an important issue in medical education and patient safety. The focus on psychotropic agents is particularly relevant, given the complexities involved in prescribing these medications. The methodology appears sound, and the study has the potential to provide valuable insights.

Is the work clearly and accurately presented and does it cite the current literature?

Yes

If applicable, is the statistical analysis and its interpretation appropriate?

Yes

Are all the source data underlying the results available to ensure full reproducibility?

Yes

Is the study design appropriate and is the work technically sound?

Yes

Are the conclusions drawn adequately supported by the results?

Yes

Are sufficient details of methods and analysis provided to allow replication by others?

Yes

Reviewer Expertise:

Human Psychology

I confirm that I have read this submission and believe that I have an appropriate level of expertise to confirm that it is of an acceptable scientific standard.

# Response

**Published:** 2024-12-14

**Authors:** Sharanya Shetty

**Affiliations:** Psychiatry, Kasturba Medical College, Mangalore, Manipal Academy of Higher Education, Mangalore, Karnataka, India

Dear Madam,

 Thank you for your valuable feedback on our manuscript. We appreciate your insightful comments and suggestions, which has undoubtedly contributed to improving the quality of our manuscript. We have tried our best to address your concerns in the modified manuscript.

1.  We have taken your feedback regarding the formatting of the results section and formatted the results section to include a table which has the common errors and areas in which the interns perform well in the form of the mean scores before and after the intervention.
2. We have also included the implications of this study under the conclusions section.

Thank you again for your valuable feedback.

 Regards

 Dr Sharanya B Shetty
